# The British Medical Association at Bristol

**Published:** 1894-09-15

**Authors:** 


### THE BRITISH MEDICAL ASSOCIATION AT BRISTOL.

### ANNUAL MUSEUM.

(Concluding Notice.)

The bookstalls offered one of the greatest opportunities of the meeting to the country practitioner. To see your books before buying them is an especial difficulty which cannot be met by an occasional visit to a large city, but here were the chief new works of many firms together on view for comparison.

Mr. Young T. Pentland showed his beautifully-printed collection by English and American authors of the highest rank. "Osler's Medicine," "Talamon's Appendicitis," Billing's great "National Medical Dictionary," Musser's new "Medical Diagnosis," the first volume of the interesting "Edinburgh Hospital Reports," "Hare's System of Therapeutics," "Dr. Prince Morrow's Genito-Urinary Diseases," and the "Journal of Pathology and Bacteriology" are among the choice books of the season, which will become constant friends in many a difficulty. He exhibited also the magnificently-illustrated "Atlas of the Diseases of the Skin," by H. Radcliffe Crocker, now issuing in parts every two months, "McClellan's Medical and Surgical Regional Anatomy," with 100 large chromo-lithographs of great beauty and value, with many others.

T. Wright and Co., Bristol, were well represented with their latest publicatious. The invaluable "Medical Annual," a perfect mine of knowledge for the busy practitioner, is said to have a circulation of 11,500 annually. "Saundby's Lectures on Bright's Disease and Diabetes," Maddox on "Prisms," and "Snells' Miner's Nystagmus," the students' series, the Essentials of Physiology, Surgery, &c., are well-known works of interest. We were much pleased with their Visiting List, compiled by Dr. R. Simpson, which is both neat and compact, and so well got up that it deserves a wide sale. Their prescription books and pocket charts are most ingenious and useful; and as a simple account book, to take the place of day book, ledger, &c., their "Jefferson's Register" is a marvellous contrivance for saving time and enabling accounts to be kept with accuracy and dispatch. We must only refer to their numerous books on nursing, and to Dr. P. Watson Williams' new work on the "Diseases of the Upper Respiratory Tract," beautifully illustrated, and supplying practitioners and students with a practical guide to the diagnosis and treatment of those diseases.

Messrs. Lewis, Gower Street, had a very large collection, among which were specially noticeable "Burnett's System of Ear, Nose, and Throat Diseases," "Buxton's Anæsthetics," and "Goodheart's Common Neuroses." A new volume of their practical series has just appeared, by Dr. Havilland

Hall, on the nose and throat, which will, we think, sustain the high character of the library. Norman Kerr's "Inebriety" is another work of extreme interest, and written by one who has made the subject a life-long study. The latest editions of "Parkes' Hygiene," "Powell's Lungs and Pleuræ," Ringer, Frederick Roberts, and many other classics were among the attractions of the exhibit.

F. T. Rebman had the largest entry in the official catalogue of any exhibitor. Many of the most elaborate and beautifully-illustrated books of the season were shown here for the first time. Starr's great work on diseases of children is probably the most complete and thorough which has yet appeared. Nothing but the great demand which exists among the united English speaking peoples for really valuable works would warrant such an expenditure as has been lavished on this book. The illustrations are magnificent, and sixty contributors have combined to portray the most recent advances made in children's diseases. The recent System of Medicine, edited by William Pepper, is another monument of Transatlantic thoroughness, and invaluable for constant reference. Robinson and Crib are bringing out an important text-book on the Adulteration of Food and Drugs and the English law relating to it. This promises to become a standard treatise on the subject. Dr. Thresh comes forward with a similar new work on Water Supplies, their Sources, Pollutions, and the Methods of Analyses. We were interested also in some well illustrated gynæcological works; among the best is "Baldy's Gynæcology," another great volume produced by many contributors, the printing and illustrations of which are of the highest order.

Ross and Co., New Bond Street, showed many of their new Eclipse microscopes for students and laboratory work, very low in price, and highly recommended by the authorities in various medical schools both here and in the United States.

Glendinning and Sons, Newcastle-on-Tyne, were noticeable for their beef and malt wine, made from Portuguese red wine, the Mosquera beef jelly, and Kepler's malt extract, a most palatable and well-blended wine food. The St. Hermes tannin wine is a remarkable ferruginous wine, imported only by this company.

Griffin and Co., Chemists, Bath, have devoted much pains to a specialty, their Nutrient Suppositories, made by an original process which is said to give the largest amount of nutriment possible in the limits of a suppository. Patients have lived more than twelve months on them alone.

The Liverpool Lint Company showed a great variety of their improved lint, bandages, and tow of very remarkable quality. The first aid dressings in packets are similar to those used by the armies of France and Germany. The transpirable vest bodices and chest protectors were extremely warm and light, and a great comfort for winter wear.

The well-known firm of John Wyeth and Brother, who claim to be the original introducers of compressed drugs, showed a beautiful array of their compressed pills, powders, hypodermic and ophthalmic discs, which were too numerous to particularise. They have some brilliant samples also of their glycerole chloride of iron, and dialysed iron, which they have brought to high perfection. Their Elixir digestive ferments are a capital addition to their series of digestive ferments, and show the usual perfection of their manufactures. Their beef juice, too, must not be forgotten.

The Bovril Company added to the samples of their famous extract, various preparations of it, such as Bovril cocoa, wine, and lozenges. Their Vril food is a preparation of milk, malt, phosphates, &c., for infants and invalids. The wild cherry sauce is a tempting relish, which might be found very useful for the table.

The Maltine Manufacturing Company exhibited their extract combined with cod-liver oil with pepsine, cascara, Easton's syrup, and also with the old favourite carrageen from Ireland.

Carnrick and Co.'s Beef Peptonsids were shown at the same stall, and prominence was given to the improved Carnrick's soluble food for infants. In this the caseine is partially digested and the milk fat largely replaced by a percentage of cocoa butter under the advice of Professor Attfield. The result is a beautiful preparation which keeps well.

Brand's essences were shown in great variety, together with their savoury meat lozenges, their concentrated broths and meat biscuits. Their Extractum Carnis and peptones are a valuable predigested food containing much nutrient and stimulating material.

In connection with these food stuffs, we should mention Allen and Hanburys' delicious Bynin and Byno-Hypophosphites, which are well taken by the most fastidious stomach, and combine great tonic properties with an excellent flavour. Their malted infants' food, their mothers' milk food, and first food for infants are widely known, and the two latter from being in the form of powder, and only requiring the addition of hot water, present many conveniences. We have already spoken of their surgical and pharmaceutical exhibits, which were of great interest.

The Anglo-Swiss Condensed Milk Company had a beautiful stall for their Milkmaid brand of milk, which contains all the cream of the original milk.

Liebig's Extract and Coleman's Wincarnis were exhibited at the stall of the Local Civil and Military Stores, Clifton.

The Bovinine Company claim not only the value of their preparation as an article of diet, but also that it has been used widely by surgeons as a local application for the treatment of indolent ulcers, which is strong evidence of the activity of its constituents.

The Liquor Carnis Company introduced their bone marrow food as an alternative to cod-liver oil. This consists of bone marrow dissolved at a low temperature, and has added to it eggs and malt extract to form a perfect emulsion. It may be found of great value in children's diseases, and even in pernicious anæmia.

The Liebig's Wine Company exhibited their pure beef juice (Vivo) and Liebig's Beef Wine with and without quinine.

Cadbury's Cocoa Company presented to visitors a beautiful volume descriptive of the manufacture and the model village where it is carried on.

Fortt's famous Bath Oliver biscuits, which took the highest award at the International Exhibition in London, 1893, were greatly appreciated, and by the same firm were shown the bottled Bath waters, the "Sulis," taken from the original springs.

Messrs. Standerwick, Stokes Croft, Bristol, had a large and handsome exhibition of cellular clothing for underwear, tennis sheets, dress skirts, &c., both in woollen and other materials. The lightness and comfort of these articles will be appreciated by those who have tried them.

T. Rogerson, 304, Brixton Road, London, had an ingenious patent vest, the Riphokam, giving greater warmth to the back without increasing the weight. This additional protection to the back is a new idea, and has been carried out with much skill. The vests have already received high commendation.

